# Does the South African government have a duty to fund influenza vaccination of adults 65 years and older?

**DOI:** 10.3389/fpubh.2024.1303949

**Published:** 2024-03-06

**Authors:** Ruach Sarangarajan, Cornelius Ewuoso

**Affiliations:** Steve Biko Center for Bioethics, School of Clinical Medicine, University of Witwatersrand, Johannesburg, South Africa

**Keywords:** influenza vaccine, solidarity, reciprocity, Afro-communitarianism, decolonization

## Abstract

In this paper, we draw on the thinking about solidarity, reciprocity and distributive justice grounded in Afro-communitarian ethics from the Global South to argue for institutions, particularly the South African (SA) government, have a *prima facie* duty to foster influenza vaccine uptake for adults 65 years and older. Although we focus specifically on the South African government to defend our position, we believe that our argument extends to all governments. Notably, these duties are that the SA government ought to make influenza vaccines freely available for the older adult in both the public and private health facilities, provided financial allocation and their extant relationships allow for this. Further, the SA government has a duty to improve influenza vaccine procurement and availability in the country, preferably through increasing manufacturing capabilities. This paper is intrinsically valuable to promote epistemic justice, thereby contributing toward the decolonization of the global healthcare system. Moreover, this project has social significance in contributing to mitigation efforts against future public health challenges associated with population aging in resource-limited developing African nations, wherein the impact of population transition will be felt most.

## Introduction

This paper draws on the norms arising from the thinking about solidarity, distributive justice and personhood grounded in the African Ubuntu philosophy and African moral philosophy more broadly to argue that institutions, particularly the South African (SA) government, have *a prima facie* duty to fund seasonal influenza vaccination of the older adult aged 65 years and above in South Africa. This will likely contribute to vaccination uptake or foster influenza vaccine access by this population group. From the outset of this manuscript, it is essential to note that although our current focus is on influenza vaccine access by the older adult in South Africa specifically, our arguments can be contextually adjusted to ground the manuscript’s thesis within other African countries. Subsequently, we believe that our argument extends to all governments. To this end, we draw on African norms that arise from values dominant in African regions.

This manuscript has become necessary since ethical reflections on *whether* governments have a duty to fund seasonal influenza vaccination for the older adult from the *unique underexplored African perspectives* are mostly missing. Existing ethical reflections on the government’s responsibility to fund the vaccination of older adults tend to adopt dominant theories from the Global North. One such position is the deontological argument that it is the government’s responsibility to fund necessary healthcare in correspondence to citizens’ right to healthcare (1). Furthermore, the older adult face specific age-related health challenges that other population groups may not experience (2). One of these age-specific health needs is prevention from influenza infection since 50–70% of influenza hospitalizations and roughly 90% of influenza-related deaths are adults aged 65 years and older (3). But vaccination programs (the most effective preventative public health measure against influenza) have been mostly aimed at infants and global vaccination coverage in the older adult is low. Miguel Kottow (4) posits that older people’s physical and health-related vulnerabilities would imply that older adults should be afforded special rights to realize these specific health needs and achieve equality through simplifying accessibility to healthcare services, especially in developing countries.

Some scholars like David Ibom and Piyush Soni (5) deny that governments have this responsibility by drawing on the principle of utility grounded in consequentialism. According to them, it would benefit the greatest number of people if hospitals were operated as businesses so that governments could allocate those health funds to other sectors. This manuscript justifies that the government is responsible for maintaining these special rights of health prevention for the older adult by ensuring they have equitable access to age-specific preventative healthcare such as influenza vaccines.

Furthermore, this project has social significance in light of the United Nations’, the Department of Economic and Social Affairs’, Population Division’s (6) estimate that the global population of those over 65 years will reach 1.5 billion by 2050. This population explosion will mostly occur in developing nations like South Africa. To effectively mitigate future public health challenges associated with population aging in resource-limited nations like South Africa, the government must prepare adequately for healthy aging through the development of comprehensive national policy in the promotion of a life-course approach (rather than only focusing on infants) to vaccination (2). This manuscript will be important in addressing the ethical considerations of influenza vaccine access for the older adult in South Africa and should contribute to more comprehensive policy formation.

In consideration of the older adult population group’s vulnerabilities to influenza as well as the impact of the burden on the healthcare system, the South African government does currently provide influenza vaccines for the older adult at no cost through the National Immunization Program 2023. This is *only* available at the countries’ public health facilities rather than in the private facilities (7). According to Statistics South (8), almost 68% of adults aged 60 and older accessed public healthcare facilities and over 31% accessed private healthcare facilities in 2021.

There are also limiting factors that undermine access to influenza vaccines, even at public health facilities. These are particularly challenging for the older adult such as prolonged waiting times, costs incurred by transport, and overburdened and understaffed health professionals (9). In fact, in 2019 (the most recently captured available data) only 67.4% of the older adult in South Africa that were surveyed were willing to consult a healthcare professional in a public health facility when ill and more concerning, 27.4% chose to self-medicate instead due to some of the barriers mentioned above to accessing public healthcare facilities (10). 31.2% of adults 60 years and older responded that they *usually* access private healthcare when ill (8). If influenza vaccines were made freely available to all older adult persons at private healthcare facilities (regardless of whether they can afford private health insurance), these challenges and barriers to accessing healthcare would significantly alleviate. Currently, at private facilities and pharmacies (such as Clicks, Dischem and Medirite) that are widely accessible by adults 65 years and above, the influenza vaccine comes at a cost, often between R109 and R250 (11). A South African study conducted in 2020 by Ijeoma Edoka and colleagues determined the cost-effectiveness of the influenza program in South Africa (which prioritizes certain vulnerable population groups) using the WHO Cost Effectiveness Tool for Seasonal Influenza Vaccination of vulnerable populations (people aged 65 years and above, pregnant women, people living with HIV/AIDS, those living with underlying medical conditions and children aged 6–59 months). The study found that the targeted vaccination program was in theory cost-effective for all the above groups except for children aged 6–59 months.

However, in South Africa, the National Immunization Program 2023 does not have the force of law and is akin to a guideline for influenza vaccine access for the older adult in South Africa exist (12). The implication is that there is a lack of willpower to enforce the guidelines. Equally, where some efforts have been made to enforce the same, it is difficult to accurately measure the success of the implementation and adherence of such guidelines since a system for adult vaccination records (other than that for COVID-19 and a paper register system for minors recording the Expanded Programme on Immunization) do not currently exist in South Africa causing barriers to efficient influenza vaccination surveillance (13), especially for specified risk-groups like older adults (14). The most recent report presenting data on vaccine coverage by age-group reported a vaccine coverage of 53% for adults 65 years and older. While this coverage rate may seem adequate, the accuracy and reliability of this data estimate may be greatly skewed due to the small sample size of 34 older adults (15). This estimate seems even more likely to be inaccurate when considering the reported statistic that only 5% of the number of doses required to immunize all vulnerable population groups in South Africa were utilized in the public sector in 2018 – just 4 years prior (16). This same study estimated the cost of vaccinating one person in 2018 was R43,61. Statistics South Africa also estimated that the South African population in (2022) included over 5.6 million older adult individuals aged 60 or older. Subsequently, we can provide an estimate that It would cost the government over R244 million to cover immunization for the entire older adult South African population (that is an additional R205 million spent on influenza immunization in the public sector compared to expenditure in 2018). Unfortunately, we could not access updated data on costs in 2023 nor were we able to find statistics on population number estimates of adults aged 65 years and above specifically. Furthermore, this estimate reflects the cost of providing free immunisastion in the public sector only and does not account for potential additional costs associated with providing it freely at a private national level as well. Our thesis that the SA government has a *prima facie* duty to make influenza vaccination available for adults 65 years and above also includes the responsibility of implementation. Additionally, we would provide clear guidelines on what concretely needs to happen to realize these duties in SA.

## Research design and methodology

This is a mostly normative ethics paper, rather than an empirical one, that draws on moral norms arising from values dominant in the Global South to address the question, “Does the SA government have a *prima facie* duty to fund the influenza vaccination of adults 65 years and above at public and private health facilities?” This approach is essential and is reckoned by others to be equally valid for research articles because of their philosophical analytic method (17, 18). Other scholars like Luis Cordeiro-Rodrigues and Kevin Behrens have also used the philosophical method we adopt. Some core sections in articles that adopt include: Introduction, Research Design and Methodology, Discussion and Conclusion. As a philosophical analytic method, the manuscript builds on relevant articles that have been retrieved from databases like PubMed, PhilPapers, and Google Scholar, using key phrases like “solidarity and African moral philosophy,” “vaccination, influenza and older adult,” “older adult vaccination and South Africa,” to name a few. For example, for our discussion on solidarity, we retrieved relevant articles from PhilPapers and Google Scholar by using key phrases like “solidarity and Afro-communitarianism,” “formulations of solidarity in African moral philosophy,” and “African philosophers and solidarity.” We were not merely interested in reading about the common features in the formulations of solidarity in African moral philosophy. We also explored the differences in formulations, especially those that might have implications for our thesis.

Our theoretical approach is vital for several reasons. First, it is crucial for epistemic justice for policies and interventions in Africa to be shaped by African values so that the communities wherein they are implemented can fully identify with such guidelines. Policies and interventions that govern people should reflect their values and be cohesive with their beliefs for people to identify with them. Second, it would lend to the acceptability of these interventions in the communities and contribute to the success of the interventions if they are guided by values already ingrained in the communities. Finally, informing vaccine interventions in an African context with values that are dominant on the continent would contribute toward the decolonization of the health system in Africa, ending scientific or health colonialism and demonstrating the exact ways normative theories from the Global South are useful and relevant alternatives to the dominant normative theories elsewhere.

To realize the set object, we draw on the moral norms that arise from the thinking about solidarity, reciprocity and distributive justice that can be grounded *primarily* in African moral philosophy and Ubuntu philosophy. We use Afro-communitarianism to encompass African moral philosophy and Ubuntu philosophy. We conceptualize Afro-communitarianism in the same way it has been described by Cornelius Ewuoso and Susan Hall (19), as the moral philosophy informed by values that are dominant on the African continent. These values are not only found in the Global South. But the thinking about these values has not come to this continent from elsewhere.

In the first section, we will describe the thinking about solidarity, reciprocity, and distributive justice in the works of African philosophers, epistemologists and anthropologists and the key values that arise from these principles. For example, a value which arises from the thinking about solidarity is that acting in aid of others can be regarded the same as aiding oneself since they are an extension of oneself as a result of the existing relationship in the community with these individuals. This way of thinking gives grounds for valuing and caring for others the same way you would for yourself. In the second section, we draw from these outlined values described in the first section to justify that the SA government has *a prima facie* duty to fund seasonal influenza vaccination of the older adult aged 65 years and above at public and private health facilities in South Africa. In the third section, we address some objections to our thesis and outline what concretely needs to happen for the SA government to realize this duty.

## Solidarity in Ubuntu philosophy and Afro-communitarianism

The term, Ubuntu, is a Nguni expression meaning humanness (20). To exhibit Ubuntu is to live a human way of life sincerely or display human excellence; to lack Ubuntu is to be deficient in human excellence (21). Thus, to exhibit Ubuntu, it is necessary to develop humanness wherein moral status, personhood and dignity are found and to lack Ubuntu is to no longer be considered a person. This begs the question, ‘How should one develop humanness?’

A foundational maxim of Ubuntu philosophy, “A person is a person through other persons” (19), roughly infers that one develops humanness through forming positive communal relationships and valuing harmony with others (22). Augustine Shutte (23) states, “Our deepest [ethical imperative] is to become more fully human by entering more… deeply into community [or harmony] with others and forgoing selfishness.” The thinking about solidarity grounded in Ubuntu requires that we conduct ourselves in a compassionate and considerate manner, that is, in a way that might benefit others. The intention behind this behavior in African thought is to care for the well-being of others (24). But to be able to show true solidarity requires acknowledging our interdependence. If we can do this, we will not feel obligated to just show compassion or try to benefit friends and family with whom we have close relations; we will equally try to benefit all other members of the community to whom we may not have personal ties but are aware that we are nevertheless connected to as a fellow functioning member within our society.

The knowledge that the well-being of others in our community is inextricably linked to our own well-being enables us to consider ourselves as a group and to act for the common good of our community and society. This way of thinking implies that we value other individuals the same as we value ourselves without needing to have personal direct ties to them because their value is found through their ability to contribute to society by their capacity to enter into relationships with others in society. Any act of aid for the greater good benefits both others around us and ourselves simultaneously. As such, there is no specific distinction between oneself and others around oneself because one regards themselves as a part of the greater community.

Contrastingly, other global conceptions of solidarity, such as that defined by Barbara Prainsack and Alena Buyx (25), which a Nuffield Council on Bioethics has used report, still lean toward a nuanced individualistic perspective with a delineation of the individuals that comprise the basis of groups and they posit that these individuals should also be regarded on an individual level, not just on a group level. This conception of solidarity does distinguish between oneself and the larger group. This conception subtlety rejects the thinking of others as an extension of oneself and may present a barrier to valuing others in the community as equal to oneself. Barbara Prainsack and Alena Buyx (25) conception of solidarity can be useful to ground for both individual and collective interests, and so it tends to be more inclusive. However, it does not account for the location of individuals’ place in communal relationships. A conception of solidarity wherein the individual and communal interest is not necessarily a dichotomy but could be considered compatible interests or where distinguishing between the two is actually irrelevant. This is also alluded to by Innocent Asouzu (26), an African philosopher who has produced numerous works in studying *Ibuanyidanda* (complementary reflection). He interestingly questions whether it is entirely necessary to categorize individualism as quintessentially Western because, in reality, both individual and communal interests inevitably exist simultaneously regardless of cultural association.

Notice that there are other ways the thinking about solidarity differs from the conception of the same in the Global North. For example, although this conception of solidarity from the Global North similarly prizes acting compassionately in aid of others, it sometimes evaluates actions in solidarity by their costs incurred. An action for the benefit of others incurring a cost implies that these beneficial deeds may become a burden or come at a disadvantage to oneself, further highlighting the individualistic perception that benefiting others does not necessarily entail concurrently benefiting oneself. Based on the preceding thinking about solidarity, solidary actions are primarily *individual-regarding*. By contrast, the African view of solidarity is other-regarding and often entails the moral duty to act for the well-being of others.

It is important to outline some conceptions of solidarity derived from common maxims and motifs in various African regions to underscore Global South’s tautology of the principle of solidarity and how it can be understood in the African context and the norms deriving from it. One foundational maxim by John Mbiti (27), a Kenyan Christian philosopher often referred to as the ‘father of modern African theology’, is, “I am because we are; and since we are therefore I am.” This maxim denotes the utmost importance of relationships with others in realizing one’s moral duties and values and developing one’s humanity or personhood. He also aptly highlights the necessity of interdependence, that one cannot exist as a human without being connected with others, and that others’ states of being are intricately bound up with our own. West African traditional Igbo philosophers (of Nigeria) often use a set of allegorical statements to draw on the principle of complementarity or mutual dependence [(28), pg. 142–148]. “Ibu anyi danda” translates to ‘no task is insurmountable for danda (a species of ants)’ [(29), pg. 11]. *Danda* can move hauls much heavier than themselves when working in mutual dependence with one another (26).

From this allegory, other African philosophers derive values of togetherness and a sense of belonging (30). In a similar vein, consider the East African Luo proverb, “Alone a youth runs fast, with an elder, slow, but together they go far” which underpins the value of togetherness, that we can accomplish much more together than we could on our own in the communal project. In this proverb, the elders provide wisdom, knowledge and guidance while, among other things, the young can offer strength and put this guidance into action. There is a mutually complementary relationship that exists with this sense of togetherness, where all parties contribute toward the communal project in their capacity but their contributions are of equal value since it collaboratively bolters the common good of those in the community. This depicts a sort of *horizontal solidarity between community members* (31). Equally, to justify how we are implicated in each other’s lives, some scholars use the motif of the Siamese Crocodile, with two heads but one stomach. This is a common motif in West Africa and it depicts how deeply connected and impacted lives are in Africa (32).

While the Global South conceptions of solidarity depicted above represent various nuanced understandings of solidarity from different African regions, it does not exhaust all possible conceptions of African solidarity. We acknowledge that within these conceptions of solidarity of the Global South remains a “missing link” of where the place of the individual can be located within the community (26). As such, the African principle of solidarity – like everything else in existence – exists in a state of incompleteness (33), wherein the space for many possibilities of enhancing and extending this principle arises. Possibly even to a conception of solidarity wherein a complementary relationship of mutual dependence between the individual and its interests and community interests can be found (26). Nonetheless, our analysis indicates that the moral imperative arising from solidarity in Afro-communitarianism often requires individuals to prize togetherness, fellowship, docility, and acting for the well-being of others.

## Reciprocity and Afro-communitarianism

Reciprocity refers to the notion that one is morally obligated to help those in their community who need aid in whichever capacity one can since others are morally required to do the same (20). A common maxim used to express this idea is that *“the right hand washes the left hand and the left hand washes the right hand.”* The moral norm that arises from this is that the relationship of mutual aid is moral, and ought to be promoted since this is who we are.

It is essential to state here that this act of mutual aid is not necessarily done *with the expectation of exchange.* Instead it is a mindset which Julius Nyerere (34) expresses aptly, “we took care of the community, and the community took care of us. We neither needed nor wished to exploit our fellow men.” Again, the African thinking of interdependence, wherein others around us are merely an extension of oneself, encapsulates this motive to act in reciprocity.

The thinking about reciprocity in the Global South is typified by the common agricultural practice in Southern Africa known as *letsema*. This is the Sesotho practice wherein members of a community undertake to assist each other during each step in farming, including ploughing, sowing, weeding and harvesting (35). Directly translated, the Setswana word *letsema* means “a group of people coming together for a common purpose” (36). This practice encapsulates several norms implicit in the significance of reciprocity in communal living.

*Letsema* calls for mutual collaboration and cooperation underpinning collective responsibility among community members (37). Furthermore, it predicates compassion in contributing toward an agricultural project that will benefit others in the community. Reciprocity is highlighted by those undertaking this practice in their recognition of the African maxims that “a single finger cannot remove fluff” and “two heads are better than one” (38). The value of collective efforts toward a communal project that brings about a common good (for those contributing as well as for others in community) is also aptly exemplified by the Setswana phrase “*kgetsi ya tsie e kgonwa ka go tshwaraganelwa*” which means “it takes collective effort to overcome a swarm of locusts” (36).

**Figure 1:**
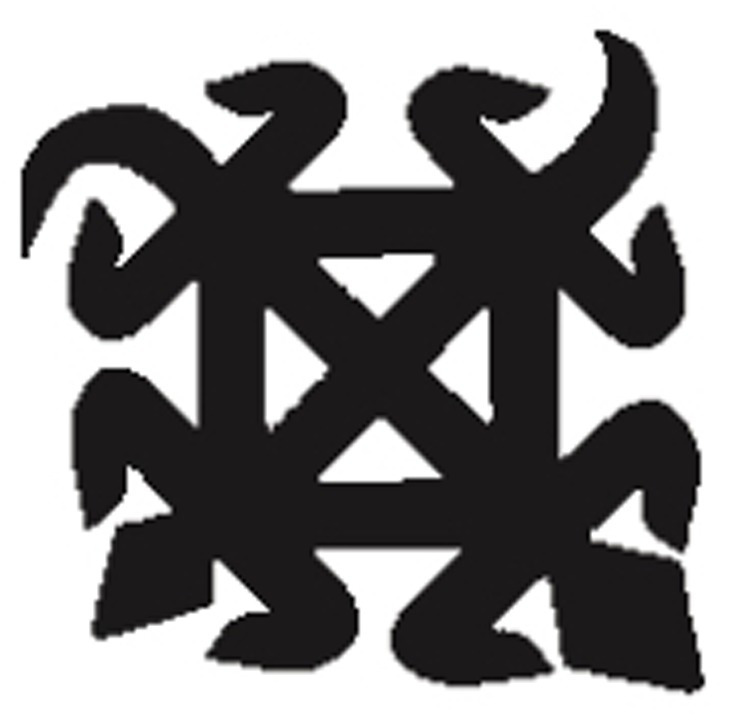
Akan symbol for *Funtumfunafu-Denkyemfunafu* - ‘Siamese Crocodiles’ illustrated by Ivana Bunuševac.

Reciprocity has also been derived from motifs from other regions in Africa. The previous section explains how the motif of the Siamese Crocodile explains the interconnectedness of lives. This Ghanian motif, *Funtumfunafu-Denkyemfunafu,* about the ‘Siamese Crocodiles’ originating from the Akan tribe is also a typology of reciprocity. The translated motif states, “Siamese crocodiles with a common stomach but struggle for food when eating” (39). This Adinkra symbol (Figure 1) depicts two individual crocodiles with separate heads and tails, but their torso is conjoined with one shared stomach (40). Although the food entering either crocodile’s mouth will come to be in the same stomach, they wrestle and compete to relinquish the flavor of the food on their own tongues and harm their survival as a whole in doing so, as they then realize (41).

In realizing that the good that is acquired by individuals in the community comes to be a shared good of the community or a common good, competing for that good is no longer necessary (42). Furthermore, preventing one from acquiring goods out of competition only harms the community. This reflects back to the needlessness of exploiting fellow community members and that aiding others in the community will help oneself in the process.

This thinking about reciprocity is not unique to the Global South and can be found elsewhere in the Global North. For example, Care Ethics also conceptualizes reciprocity as mutual aid. However, the mutual freedom to enter a reciprocal exchange is necessary and requires a mutual agreement to this exchange. A response to reciprocal action by one party (which may be unequal) is then demanded by the other party to the agreement (43). This is not necessarily true in African thinking. For the reason that we are already in existing potential reciprocal relationships with everyone else with whom we are in the community. In other words, there is no specific agreement between parties to enter into a reciprocal relationship as such. Moreover, acts of goodwill to others in the community are done neither with the expectation of receiving anything in exchange nor to require an immediate reciprocal response of equal measure from others (34), as it tends to be the case with Care Ethics.

## Distributive justice and African moral philosophy

Justice alone entails relating to others in a right manner wherein each person is given their due (44). Distributive justice in the scholarship on Ubuntu requires one in a state of authority to equitably distribute advantages and disadvantages accordingly to reach as close to a state of equality among disparity groups as possible (45).

Although distributive justice is not uniquely an African principle, there are unique features of this principle emanating from the literature in African philosophy that are worth highlighting. First, distributive justice is sometimes differentiated from commutative justice. Both distributive justice and commutative justice are considered as expressions of social justice in the literature on African (moral) philosophy. While distributive justice describes first-order duties of institutions and states to their citizens (to protect their civil liberties, distribute goods equitably and create a conducive environment for communal relationships), commutative justice describes the responsibilities of citizens to one another and the State. Notably, their responsibility to be solidary to one another and to the State (46).

Evidently, commutative justice also involves distribution of some sort, but this is a second-order duty that explores issues around equity and relations on the horizontal (among citizens or equal parties) and vertical (toward the State). For example, this conception of justice can enhance our thinking about citizens’ duty to pay taxes or vote in elections. Contrastingly, distributive justice describes the State’s responsibility to their citizens.

Second, although *social justice* and *distributive justice* are conceptually distinct, nonetheless, it is not uncommon to find that the discussion on distributive justice is sometimes framed as *social justice*. Specifically, matters of *social restorative justice* in Africa, such as land redistribution to rectify unjust colonial land distributions, have been reframed and understood as *distributive justice* in some publications (47). For example, Thaddeus Metz (48), one of Ubuntu’s most prominent African philosophers, does not distinguish between social justice and distributive justice. He contends that Ubuntu philosophy bears many values reminiscent of social justice, such as respect for all, communal participation and societal inclusion. Ubuntu philosophy, he adds, is also representative of distributive justice wherein values of equity, through a culmination of collective responsibility and promoted interdependence, and respect for others, through caring about the wellbeing of others in the community (solidarity) as a motive to restore equality are located. In other words, the values found in Ubuntu are positioned as expressing core concerns about social *or* distributive justice.

Furthermore, in the scholarship of African authors who contend that a distinction ought to be made between distributive justice and social justice, it is not uncommon for one to read the following to be the core of distributive justice from that positionality; (i) it entails the responsibility of States and established organizations to honor the rights of individuals, including their health rights, (ii) to create opportunities for individuals to enjoy a deep communal relationship, which may include funding their health care since illness can undermine their to enjoy communal relationships, and finally, to regulate interactions among individuals (46).

Although the main aim of this section is evaluative rather than descriptive, it is worth outlining that distributive justice in the African moral philosophy literature broadly. Ubuntu philosophy, in particular, requires governments and institutions to showcase humanity to their citizens by ensuring that they have a decent minimum to flourish, *viz.*, they can access the basic conditions necessary for participating in communal relationships or share a way of life with others (44).

In the subsequent section, we demonstrate how this will require governments to fund the vaccination of their older adult population, particularly in private healthcare facilities. Notably, suppose communal relationships (and/or the capacity for the same) are the basis of morality and moral status in the African Ubuntu philosophy. In that case, an essential way of fulfilling the duty of distributive justice is for governments and established institutions to remove conditions that undermine participation in communal relationships, especially when they can. Illness undermines participation in communal relationships. To understand how, notice that one needs to be a subject and object of a relationship to have full moral status in Afro-communitarianism. To be a subject is to be able to commune with others, exhibit caring or other-regarding behaviors toward others. Objects of communal relationships are those with whom one communes. Illnesses undermine one’s capacity to be *subject* of this relationship since it reduces one to an *object* of others’ care, love and compassion.

Notice that we have not claimed in this section that all sick people cannot exhibit caring relations toward others at all. Sicknesses and illnesses have a spectrum, and individuals may still be able to exhibit other-regarding behaviors to others, even in that state. Instead, we focus on the more intense forms of sickness, which are often lethal, like seasonal influenza in the older adult. We contend that these often undermine adults 65 years and above’s capacity to enjoy deep communal relationships as *both* a subject and an object of these relationships. As we demonstrate, since governments have a responsibility to alleviate conditions that undermine citizens’ capacity. In that case, they ought to fund the influenza vaccination of this population group. The preceding is, in fact, a moral response to the rights adults 65 years and above enjoy as a party in communal relationships with the government. In other words, communal relationships encumber. Thaddeus Metz aptly expresses this when he remarks that, “if one has been party to a communal relationship with others [such as the government]…. then one can have some strong moral reason to aid these intimates as opposed to strangers, even if the latter are worse off and if one did not promise to aid the former” [(49), pp. 44]. The basis of a State or government’s duty of distributive justice to others is communal relationship. We provide further justification in the subsequent section.

## Government’s responsibility to fund influenza vaccination

In the previous section, we provided an overview of – and described the moral norms that can arise from – the principles of solidarity, reciprocity and distributive justice grounded in Afro-communitarianism broadly. Furthermore, we differentiated these conceptions from the thinking about the same in the Global North and compared various other conceptions of the same in the Global South. It is important to note that solidarity, reciprocity and distributive justice do not exhaust all the principles in the African (Ubuntu) philosophy. There are others like identifying with others. Nonetheless, these outlined principles are relevant to this section’s evaluative goal. Equally, many other conceptions of solidarity, distributive justice and reciprocity globally are not represented in this paper but are no less critical in their applications in ethics broadly.

This section draws on the moral norms articulated in the previous section to justify why governments broadly, but the SA government in particular, have a *prima facie* responsibility to fund seasonal influenza vaccination of the older adult in private and public health care facilities. To enhance the public health importance of this manuscript, we also describe what efforts are required to ensure that such vaccines are *available* and *affordable.* Notice that we do not contend that accessibility issues *only concern* availability and affordability since such issues will also include concerns around *acceptability*. Nonetheless, we focus on availability and affordability in this manuscript and defer the discussion on acceptability for another manuscript.

To justify our position, notice that most older adult Africans are unemployed, and few receive a small pension fund or government grant, which is just enough to cover their living expenses. The situation is worse for older people in South Africa. A 2022 study shows less than 15% of adults aged 60 and older in South Africa are employed (50). Precisely, in South Africa in 2020, BankServAfrica (51) estimated under 19% of adults over 60 years old receive private pensions (some of which receive less than R6510 per month and, thus, fall under the qualifying threshold for social grants as well) and under 70% receive Old Age Grants (OAG). This is consistent with the abovementioned study that shows 69% of the older adult receive an OAG of only R1780 (50). BankServAfrica (51) also found that under 8% of adults over 60 were business owners or still employed in 2017. This leaves an estimate of over 6% of adults over 60 years old with no income, pension fund or government grant (including those with no income from partners or spouses) in South Africa (51). In the current climate in South Africa where unemployment has increased to about 32%, these individuals are vulnerable financially and physically, given their advanced years. Notably, many of them cannot work or procure income for themselves or easily attain free quality and adequate basic healthcare without aid. Physical and mental declines in this age group present further barriers to accessing healthcare services.

Moreover, 69% of older adults receiving only the OAG would fall far short of a “decent standard of living” according to SASPRI, the Studies in Poverty and Inequalities Institute, and the Labor Research Service (52). SASPRI contends that R7541 per person per month equates to a “decent standard of living” in South Africa in 2020. For argument’s sake, say that 19% of adults receive private pensions of R6510, and all private pension owners also receive OAGs of R1780. In that case, about 50% of older adult citizens would receive only R1780 per month (not considering the number of older adult individuals that do not own their own housing and have to pay rent or individuals that live with other families). This means that over 56% of the older adult (including those with no income) would have a low standard of living and experience barriers to a good quality of life including accessibility to basic healthcare services.

Indicatively, low economic levels can significantly impact other quality-of-life factors such as household services and health. With 36.4% of the older adult living in households of three or more generations (50), overcrowding can become a devastating health factor during seasonal influenza outbreaks. A low economic status can also affect accessibility to quality healthcare services through barriers of transport costs and long waiting times at public facilities (9).

Although the manuscript focuses on adults 65 years and older, given that (i) this is the retirement age in South Africa, and (ii) adults 65 years and older tend to be more vulnerable than adults younger than 65 years. Nonetheless, it is worth stating that influenza vaccination for adults 60 years and older falls under basic healthcare and is a core requirement of what could foster the flourishing or well-being of this population group. This is because of the high risk of hospitalizations and mortalities influenza poses for this population group. Preventative healthcare, like vaccines for adults aged 65 years and older, should also be considered basic healthcare since it is often life-saving medical care. According to the American Medical Association (53), basic healthcare includes that which protects the most vulnerable population groups and, specifically, affords those that are historically disadvantaged (in this case, the older adult has been marginalized in preventative care, with curative and palliative care being the dominant alternative) with special care.

Suppose governments have a duty of distributive justice to their citizens to provide the essential minimum for their flourishing. Equally, suppose preventive healthcare, particularly seasonal influenza vaccination of adults aged 65 years and above, constitutes this population’s basic health requirement. In that case, the government has a responsibility to fund this care for this population since, in fact, many individuals in this population group often struggle with a low standard of living. Accordingly, the government ought to make influenza vaccination accessible to adults in this group, even in private health care facilities. Notably, the older adult comprise one of the high-risk population groups (with lower immune systems).

For this reason, the requirement of distributive justice implies that governments should afford them a special minimal healthcare service to fulfill their specific health needs. This will be an appropriate moral response by subjects of communal relationships (the government) to the objects (adults aged 65 years and above) of these relationships since it is a crucial way of acting to improve the latter’s life quality. Precisely, the moral imperative of distributive justice that can be grounded in communal relationships is that a party in this relationship ought to be willing to go out of their way to assist the object of this relationship to flourish, especially when the subject can. As a party in communal relationships, adults aged 65 years and above are also entitled (that is, they have a right) to be aided or supported by others since *communal relationships encumber.* Notably, “where there is some relationship, there are some obligations” [(44), pp. 4]. With certain rights held by this vulnerable population exist corresponding responsibilities by other parties (usually stronger parties or those in authority) to maintain, protect or create an environment for realizing these rights.

There is another justification – grounded in the thinking about reciprocity – for the claim that governments are responsible for maintaining and protecting these corresponding rights by ensuring influenza vaccines can be accessed by adults aged 65 years and above at no cost, including at private health facilities. Specifically, adults 65 years and above have contributed to society over the years. Equally, the older adult, including adults 65 years and above, are highly revered in many African communities. Both their age and life experience position them as conveyors of knowledge and moral education essential for youth formation. This is why the death of an older person is often considered a huge loss to the community (54). There are other ways adults aged 65 years and above have also contributed to the State, such as through tax contributions. These contributions entitle them to receive the government’s support in realizing their basic (medical) needs. It is important to note that older adult individuals do not necessarily share their wisdom and educate younger generations merely because they expect a reciprocal act of care but because out of reciprocity, aiding others is a duty. What this means is that the older adult are aware that their acts of aide will be reciprocated in time – that they will be cared for like they have cared for others – but this is not necessarily what motivates the acts of aid/care. Rather, the older adult are aware that the wellbeing of others in the community are inextricably bound up with their own. Out of this knowledge springs the duty of care and aid which is carried by everyone in the community. Subsequently, one need not be afraid of being exploited through unreciprocated acts of aid and can rest in the fact that they will be adequately taken care of in their time of need. The act of care is out of compassion and responsibility for the wellbeing of others in the community and not necessarily because one expects a reciprocal act to repay this debt. However, it does provide a good motivation to avoid selfish acts. In the context of our argument, if governments do not provide freely available influenza vaccines for the older adult, it does not necessarily mean the older adult will stop sharing their wisdom and knowledge. But, rather this means that governments are not fulfilling the reciprocal duty of taking care of the older adult’s medical needs which they now ought to do.

About medical health needs (such as vaccines), it is worth stating that the South African government sometimes subsidizes basic health services for vulnerable populations (often for those who are financially vulnerable like students and pensioners) to ensure any vulnerable person, no matter their background or circumstance would be able to afford and access the service to meet their health need. As previously noted, the government does this through public health facilities. However, this section contends that the government ought to make this opportunity available at private health facilities. Concretely, the section contends that the government has a responsibility to maintain and preserve the special rights of the older adult to life-saving preventative healthcare by ensuring that influenza vaccination is available *at no cost* for this older adult population at public and private health facilities.

One advantage here is that this position will have a secondary effect of promoting public health. Although there is a lack of data on vaccine effectiveness on the South African older adult population due to a lack of Randomized Control Trials, a study conducted in the United States found that the 2019–2020 influenza vaccine was associated with a 41% decrease in the risk for influenza-related hospitalizations for older adults (55). Notably, increasing influenza vaccine coverage for the older adult could significantly reduce the number of influenza-related hospitalization and greatly reduce the burden on the healthcare system and saving on limited resources in resource-constrained African nations.

Furthermore, suppose countries signed on to the WHO Global Influenza Strategy are serious about reaching the strategic goals of reducing the seasonal influenza burden, controlling the risk of zoonotic influenza and acting in preparation to alleviate the impact of influenza pandemics. In that case, they ought to coordinate their behavior to be in line with meeting these goals by ensuring seasonal influenza vaccines are affordable most of all to the most vulnerable population groups, in this case, adults aged 65 years and older since this group has the highest influenza-related mortality and infection rates. They would need to coordinate their behavior and collaborate in the communal project of fostering influenza vaccine uptake in adults aged 65 years and older, expressly by making vaccination available to this population group. South Africa and all other WHO countries have a collective responsibility to achieve global health. For this to be realized, each country must act accordingly, forsaking selfish acts that might only benefit their own country in the short-run and bolstering compassionate, collaborative acts that would benefit all countries (health) in pursuit of this common goal. This is what it means to exhibit solidarity with the citizens and other countries. Notably, this derives from the interconnectedness of lives: the health of SA is deeply interlinked with the health of other countries and global health, in the same way that the health of citizens can have great implications for society. Suppose, as we have demonstrated in a previous paragraph, that the older adult perform essential roles in fostering the moral formation of the youths. In that case, the SA government ought to foster their basic health needs since health is required to perform this task. Also, suppose the older adult citizens are an extension of the government as valued community members. And if it is true, one should value others in the community the way one values himself. Then in that case, it is the government’s prerogative to act within their power to preserve the valuable lives of older adult individuals in the community by ensuring that influenza vaccines are available. In doing so, the government would be identifying with older adult citizens by seeing themselves together with the older adult as part of a whole, by acknowledging the older adult as an integral part of the community as government leaders themselves are. Furthermore, the government would be exhibiting solidarity with the community by fulfilling the duty to ensure influenza vaccines are available and playing their part in fostering the uptake of influenza vaccines for the older adult.

By both exhibiting solidarity with vulnerable citizens (caring for their well-being in a considerate manner and acting to benefit citizens); equally by coordinating behavior to meet the WHO Global Influenza Strategy goals in identifying with other countries, thereby protecting these vulnerable populations from influenza infections and its complications, the SA government would be exhibiting solidarity or forming harmonious relationships with adults aged 65 years and older. By establishing and maintaining harmonious relationships in this way, the government would also develop personhood as they would become even more valuable in their ability and willingness to relate harmoniously with citizens.

Furthermore, suppose the global community wants the older adult to vaccinate against influenza to reduce mortality rates. In that case, influenza vaccination ought to be made available to them. It seems counter-intuitive or irrational to require the older adult to vaccinate against influenza but it fails to make vaccination easily accessible. Funding vaccination will be an important way of making it accessible since most individuals are pensioners, retired or unemployed. In this sense, the older adult are also vulnerable because they do not have the full financial ability to address their health needs, including the basic ones.

Finally, although the duty that this section defends is only a *prima facie* duty, implying that this duty must be weighed against other obligations that might be more important. Specifically, neither the moral imperatives that arise from the thinking about distributive justice nor solidarity/reciprocity imply that the duty this section defends is an absolute one. Contrary to consequentialist moral theories that require maximizing consequences, the African Ubuntu philosophy requires one to aid others or exhibit solidarity toward them while considering how one’s extant obligations might be impacted. This is not to say that consequentialism is not effective as a moral theory in the normative application of public health dilemmas such as this. Rather, what we are emphasizing here is that while both normative theories can consider the impact of extant governmental obligations, consequentialism requires one to be impartial which is converse to the essence of solidarity. Subsequently, duties in consequentialism are borne out of the greatest potential positive/beneficial outcomes of fostering those duties, but Afro-communitarian duties are bound up in compassion for the needs of others. As such, there is a duty to meet the greater comparative (to other less vulnerable population groups) needs of minority and vulnerable groups through more urgent aid and greater attention to care, regardless of the public health consequences of not meeting their health needs. Afro-communitarian holds at its core, the value of distributive justice.

It is also worth emphasizing the academic importance of drawing on Afro-communitarianism. Particularly, the academic significance of our approach is that it contributes to epistemic justice so that future public health policy and policy reformation *in Africa* be informed by African values rather than by Western values which are sometimes in direct contradiction to their own (African) values as we have demonstrated above. Moreover, individuals are more likely to accept policies that align with their values.

Concretely, the SA government’s primary function to protect life and property would imply that a government ought to fund influenza vaccination for the older adult unless doing so will significantly undermine this primary responsibility. For example, during a time of national crises (wars or pandemics to name a couple), say in this case the country would experience adverse public health outcomes if the health of injured soldiers in a war or frontline workers during a pandemic were not prioritized over health expenditure on vaccines for the older adult. In that case, the extant obligation to prioritize funding allocation to these more dire health needs would outweigh the duty to provide freely available influenza vaccines for the older adult during that time. It is impossible to be able to predict what these extant obligations might be. Moreover, governmental duties and priorities would vary greatly between different African nations. Subsequently, in our argument, we do not limit the potential governmental obligations to any such confined list but rather leave it open to maintain flexibility and adaptability between African countries. However, suppose a government could easily fund the health care needs of the older adult but fails to do. In that case, it disrespects the older adult, the object of communal relationships.

## Institutions’ responsibility to ensure availability of influenza vaccines

In the preceding section, we demonstrated how the principles of solidarity, reciprocity and personhood arising from African philosophy provide grounds for the government’s *prima facie* duty to ensure influenza vaccinations for older adult South Africans are free of cost in both public and private sectors to increase ease of accessibility. This section will describe what needs to happen to fulfill this duty.

In the context of influenza vaccine uptake strategies, we must address the dimension of availability in two-fold. First, referring to the availability of the vaccine in terms of supply meeting needs and second, the availability of vaccines to the older adult in locations in which the older adult most often access immunization services. In addition, it is equally important to consider the existing surveillance services’ capacity to gather data to measure, monitor and evaluate the success and challenges of the proposed vaccine promotion strategy. Concerning the former, although a reported 14 out of 31 African countries have influenza vaccines available (14), the Global Alliance for Vaccines and Immunization (56) white paper report found a gap (between demand and supply) in the African vaccine market. To address this, there is a need for influenza vaccine manufacturing in African countries (57). Eight African countries have vaccine companies operating with just four of these facilities currently manufacturing vaccines at the time of this report and only one of which (located in Morocco) has been reported to handle influenza vaccines for importation, but is not involved in its manufacturing (58). There is a glaring need for the development of influenza vaccine manufacturing capacity in South Africa which would ultimately significantly relieve the burden of costs of vaccine import and could lessen the impact of transportation disruptions in the supply chain, thereby increasing availability in the long run. In addition to increasing vaccine manufacturing capacity in South Africa, research and development of influenza vaccines in South Africa, as well as addressing research-related ethical concerns such as funding and the ability to conduct randomized controlled trials with the older adult. For this, greater collaboration between the government and academia will be required.

Concerning the latter, it is the government’s responsibility to ensure vaccines are made affordable for those with the most health need and the least able to afford it where they can access it.

It is important to consider where most communities in African countries access immunization and other health services to foster the uptake of influenza vaccination among different population groups broadly or tailor vaccine promotion strategies accordingly. A study has found that both children and adults access pharmacies for immunization services (59). In South Africa, a reported 7 out of 10 households choose to access public clinics or hospitals if a member needs medical care (60). One way to ensure vaccination accessibility in light of this manuscript’s thesis is for government to address the barriers to the same that we mentioned in the previous section. Furthermore, the public healthcare system in South Africa consists of 422 hospitals and 3,841 clinics or health centers, which indicates how much easier access to health clinics/centers is for most communities than hospitals in terms of distance (60).

Mobile clinics may also be utilized. Mobile clinics are vehicles that have been refurbished to provide clinical services in remote locations such as rural areas and can provide vaccinations as well (61). In Kenya, these mobile clinics have a context-specific alternative to a motor vehicle – they use camel mobile clinics to travel to remote desert areas (62). SA government must adapt these clinics to the contextual reality of her people.

Keeping medical records of patients at each facility where individuals access vaccination (including records from mobile clinics) will enable an estimate of the required annual supply of influenza vaccines for each facility to cover adults 65 years and older. This can be done by assessing the number of older adult patients that have accessed each facility yearly and eliminating duplicates across facilities to ensure each access point has an adequate supply available for older adult citizens to vaccinate against influenza before influenza season each year. This strategy will eliminate waste and ensure the supply will meet the potential demand in a way that is easily accessible for the target group.

Additionally, this kind of geographical information would be useful in the formation of monitoring and evaluation of implementation strategies. This surveillance reporting must be upscaled so that both successes and challenges of strategies can be picked up and measured. It is also important to note that in some African countries, vaccination record systems for adults (other than for COVID-19) do not currently exist (13). Databases wherein health records of the annual number of influenza vaccines administered to adults aged 65 years and older at various service providers would be useful to measure the success of influenza uptake strategies that are implemented and in further age-specific influenza research studies as well.

## Potential objections

This section explores some potential objections to the argument presented in this manuscript. Notably, a critic could contend that requiring the South African governments to provide influenza vaccines freely could spiral into forms of authoritarianism. The government may think that they have the responsibility to dictate their citizens’ health habits or choices. The Chinese one-child policy is one example of how the position we endorse may encourage governments to make arbitrary health, including reproductive decisions for their citizens. Adults 65 years and above who exercise their freedom to refuse vaccination may be penalized or sanctioned. Freedom may be curtailed in the world where governments believe they have the prerogative to make health decisions for their citizens.

In response, notice that the position of this section is not that freedom *ought not* to be curtailed. While freedom of choice in health decisions is often important, the greater duty to foster overall public good or harmony (as we have seen with various forms of restrictive measures during COVID-19 outbreak) may require governments to limit an individual’s right to freedom. Nonetheless, the counter-argument that this perspective could lead to an authoritarian government is a valid concern. However, we do not think this is necessarily warranted. This is for two reasons. First, authoritarianism will necessarily involve coercing individuals to act in certain ways. However, coercion entails acting in unfriendly ways toward another from the Afro-communitarian perspective (63). This is justified only if it is necessary to end similar unfriendliness. Exhibiting unfriendliness toward those who have not been unfriendly will be a failure to share a way of life with them or exhibit other-regarding behaviors toward them. Coercing individuals in certain ways when they have not been unfriendly will be a failure to exhibit solidarity toward them. This is what is entailed by authoritarianism. Specifically, this response demonstrates that suppose governments act in authoritarian ways toward their citizens. In that case, this would not be a consequence of the philosophy this section draws on since – from this positionality – one ought to be friendly to those who have been friendly and unfriendly to those who have been unfriendly. Yet authoritarianism often entails acts of unfriendliness toward those who have been friendly.

Second, this concern is also unwarranted due to an evident disconnect. Particularly, defending a health intervention that requires the government to take the financial responsibility to increase accessibility by ensuring a vaccine is accessible to a population does not necessarily afford the government the authority to coerce the older adult to access these vaccination services involuntarily. Ensuring the vaccine is available to the older adult does not guarantee that these individuals will access it, but rather this action serves to alleviate an important barrier to accessibility for those who are voluntarily willing to accept the vaccine. This manuscript highlights that the government has this financial responsibility to ensure decent minimal healthcare is accessible since they have been given the authority to provide for such services, for example, with citizen’s taxes. However, it is difficult to see how this responsibility implies that governments *ought to* limit all rights to freedom of choice in healthcare by individuals. As previously stated, limiting individuals’ health decisions is permissible on the condition that this is necessary to end comparable unfriendliness. However, arbitrary health decisions would not be ethically justifiable because they would neither be considered a necessary measure in ensuring decent minimal healthcare for all nor would these actions garner harmonious relationships between the government and its citizens. In fact, directing arbitrary health decisions for citizens would harm relationships between the government and its citizens and could cause civil social tensions, as observed during the one-child policy in China (64).

Another critic may express doubts that my contribution will have the impact that it intends. For example, what needs to happen and at what level to realize the duty to fund the influenza vaccination of the older adult, particularly in South African private healthcare facilities? Suppose there are challenges or difficulties with accessing vaccination in public health facilities. In that case, what concrete changes need to occur to make influenza vaccination more accessible? It seems – to move from rhetoric to action – fine details concerning *how* the government can realize this duty (beyond merely claiming that they ought to fund) need to be outlined carefully and intelligently, and I have not done this to a significant degree.

To some extent, we have partly answered this question in a previous section. Nonetheless, we acknowledge the concern that this position can be construed as idealistic and rhetorical. However, we argue that concrete moral stances on how to increase accessibility ethically are important primary conversations to open in the discussion of increasing influenza vaccine uptake by the older adult in South Africa. The overall project of increasing influenza vaccine uptake is an ambitious, albeit not impossible, one to undertake. As such, in this manuscript, we mostly endeavor to provide a conceptual exploration of existing structures and barriers to accessibility by examining the availability and affordability dimensions of the influenza vaccine. In this regard, we acknowledge that the collaboration between the government, pharmaceutical companies and academia that we suggest in the previous section will be insufficient to foster uptake. Vaccines may be freely available and adults 65 years and above may still refuse to vaccinate. We will address this question in a future manuscript.

This manuscript provides a moral foundation for future conversations on how policy reformation should be grounded in African ethical considerations. However, this manuscript does not set out to outrightly propose how these policy changes ought to take place. Our proposal in this manuscript may be in a state of incompleteness, but it does not render our argument irrelevant or unnecessary by any means because it is, in fact, the very crucial first step of many in directing and informing future public health policy formation as many conceptual bioethical arguments are wont to do. Its very existence in incompleteness allows for a space where ethicists, policymakers, stakeholders and financial advisors can collaborate, transform and advance our contribution.

Notwithstanding, our manuscript brings to the forefront a significant equity problem in preventative health measures the older adult population faces in South Africa and calls for further discourse on where to go from here to address this problem. Specifically, while we argue that the government ought to fund influenza vaccines for the older adult in both private and public healthcare facilities to overcome accessibility barriers in the public domain and to increase the availability of vaccines to the target population, we do not pretend to have all the answers regarding the concrete implementation of this position. The intricacies of exactly how public and private funding ought to intersect or the amount of government interference in the private health sector to realize this objective must be worked out. Nonetheless, we believe that the direct cost of influenza vaccination of the older adult at private health facilities ought to be communicated directly to the government, who should defray this cost from the annual health project. The annual health budget may need to be expanded to accommodate this cost. Our argument also somewhat supports a mixed public-private funding landscape in South Africa that aligns with the vision of the National Health Insurance. Further research on financing structures for the availability of the influenza vaccine for the older adult at private facilities ought to be conducted.

Another critic may also point out that there are *far more important* ways for governments to support the older adult to meet their basic health needs beyond funding vaccination. For example, adults 65 years and above often suffer *more* from old-age-related diseases not limited to neurodegenerative and cardiovascular diseases. The focus on influenza vaccination seems to distract attention away from these health burdens.

This is quite an important and valid concern. While we acknowledge that non-communicable diseases are a great health burden on the older adult population and deserve attention and funding, we do not think there needs to be a dichotomy in choosing which health challenge deserves attention. We argue that this critique presents a false dilemma that the government cannot focus efforts on both communicable and non-communicable diseases and that this view, even if it may be true, does not necessitate avoiding discussion over preventative health methods for the older adult.

Even operating under the guise of the conditions of this premise that resources are so limited that focus should be given to either, we argue that this intervention is not a waste of resources and is in fact, using resources more sustainably. This argument is substantiated by numerous studies reporting the cost-effectiveness in targeting vulnerable populations for influenza vaccination, calculated by the number of influenza cases averted, hospitalizations and deaths averted and cost per quality-adjusted life year (65, 66). Encouraging vaccine uptake for the older adult will also be important in reducing anti-biotics and anti-virals prescribed as well as possibly decreasing nosocomial infections without adding to the burden of life-long care costs, which plays a factor in the cost-effectiveness of the vaccine, a significant consideration in resource-constrained countries especially (67).

Although it is difficult to calculate the estimated cost burden averted, South Africa particularly lacks age-specific data collected by their three respiratory illness surveillance teams – Viral Watch influenza-like illness, influenza-like illness, and pneumonia surveillance (68). Furthermore, cost-effectiveness varies yearly according to several factors, such as the burden of HIV infection in the country, the type of influenza strain circulating, and the vaccine type (69). Nonetheless, we believe that preventative healthcare for adults 65 years and above will positively impact the collective ability to limit the impact that non-communicable diseases will have on them.

Finally, a critic may point out that even if a government provides free vaccines and the older adult reject the same, our argument will still not have the impact we hope it would have. The older adult also need to accept to be vaccinate for vaccination programs to be effective. Viruses are controlled when the majority of the population has either been exposed to the virus naturally, or through vaccinations. The smaller the percentage of the population that acquires immunity, the more hosts that the virus can infect. Thus, does the government also have an obligation to mandate vaccinations.

This is indeed an important question. Vaccine access tends to encompass three issues, (i) affordability, (ii) availability and (iii) acceptability. In this current manuscript, our primary focus is to address governments responsibility to make vaccines free to address the questions around affordability and viability. In a different manuscript, we draw on the key norms arising from incompleteness and conviviality in African scholarship to justify the moral responsibility of the older adult to vaccinate against influenza to address key questions around acceptability.

## Conclusion

This primarily normative and theoretical paper has drawn on moral norms from the Global South, namely solidarity, reciprocity and personhood grounded in African philosophic thinking. In this argument, we addressed two main considerations surrounding the accessibility of influenza vaccines for adults 65 years and above in South Africa. Firstly, we claimed that African governments have a *prima facie* duty to make seasonal influenza vaccines free to the older adult. Secondly, we asserted that institutions (including the government) have an obligation to ensure influenza vaccines are available in respect to vaccine procurement and distribution at frequently accessed locations by the older adult. Finally, we responded to some potential counter-arguments on unintended consequences of the government holding power over arbitrary health decisions, that our argument lacks a concrete foundation to move from rhetoric to action and that other more important health concerns regarding the older adult ought to be advocated for instead of influenza vaccine uptake.

It is important to note that this paper forms part of a series and has only addressed the affordability and availability aspects of influenza vaccines for the older adult. We acknowledge that accessibility is also significantly contingent upon the acceptability of the vaccine. Subsequently, the next paper in this series seeks to address these ethical considerations of acceptability by focusing on factors such as cultural and traditional values, attitudes, beliefs and social norms held by the older adult population in South Africa. This is an important step in our project because only once we have comprehensively addressed all three dimensions of accessibility can we propose a more concrete framework with which to direct public health policy to drive a more actionable argument.

## Data availability statement

The original contributions presented in the study are included in the article/supplementary material, further inquiries can be directed to the corresponding author.

## Author contributions

RS: Conceptualization, Writing – original draft, Writing – review & editing. CE: Supervision, Writing – review & editing.
